# Volatile vs Total intravenous Anaesthesia for major non-cardiac surgery: a pragmatic randomised triaL (VITAL)

**DOI:** 10.1186/s13063-024-08159-w

**Published:** 2024-06-27

**Authors:** Joyce Yeung, Shaman Jhanji, John Braun, Janet Dunn, Lucy Eggleston, Samuel Frempong, Louise Hiller, Claire Jacques, Monica Jefford, James Mason, Ramani Moonesinghe, Rupert Pearse, Benjamin Shelley, Cecilia Vindrola, on behalf of VITAL trial team

**Affiliations:** 1 The Royal Marsden NHS Foundation Trust, London, UK; 2 Warwick Clinical Trials Unit, Warwick Medical School, University of Warwick, Coventry, UK; 3 Centre for Health Economics at Warwick, Warwick Medical School, University of Warwick, Coventry, UK; 4 University College London, London, UK; 5 Queen Mary University of London, London, UK; 6 School of Medicine, University of Glasgow, Glasgow, UK

**Keywords:** General anaesthesia, Major surgery, Patient outcomes, Post-operative complications, Randomised controlled trial

## Abstract

**Background:**

Improving outcomes after surgery is a major public health research priority for patients, clinicians and the NHS. The greatest burden of perioperative complications, mortality and healthcare costs lies amongst the population of patients aged over 50 years who undergo major non-cardiac surgery. The Volatile vs Total Intravenous Anaesthesia for major non-cardiac surgery (VITAL) trial specifically examines the effect of anaesthetic technique on key patient outcomes: quality of recovery after surgery (quality of recovery after anaesthesia, patient satisfaction and major post-operative complications), survival and patient safety.

**Methods:**

A multi-centre pragmatic efficient randomised trial with health economic evaluation comparing total intravenous anaesthesia with volatile-based anaesthesia in adults (aged 50 and over) undergoing elective major non-cardiac surgery under general anaesthesia.

**Discussion:**

Given the very large number of patients exposed to general anaesthesia every year, even small differences in outcome between the two techniques could result in substantial excess harm. Results from the VITAL trial will ensure patients can benefit from the very safest anaesthesia care, promoting an early return home, reducing healthcare costs and maximising the health benefits of surgical treatments.

**Trial registration:**

ISRCTN62903453. September 09, 2021.

**Supplementary Information:**

The online version contains supplementary material available at 10.1186/s13063-024-08159-w.

## Introduction

### Background and rationale {6a}

More than 1.5 million major non-cardiac surgeries are performed in the NHS each year, including a variety of procedures from cancer resections to orthopaedic surgery [1]. High-quality general anaesthesia is essential for patients undergoing major surgery. In the NHS, general anaesthesia is most often maintained with an inhaled volatile anaesthetic agent (e.g. sevoflurane, isoflurane) [2]. A commonly used alternative is to maintain anaesthesia using infusions of intravenous anaesthetic drugs (e.g. propofol, remifentanil), a technique termed total intravenous anaesthesia or TIVA. The two techniques have important differences in side effect profile [3, 4]. There is a distinct lack of data describing the benefits and harms of either technique in terms of important patient outcomes.

Whilst the clinical endpoint of general anaesthesia is broadly similar between inhalational anaesthesia and TIVA, their underlying pharmacological actions are very different. Propofol (an intravenous anaesthetic drug used to provide TIVA) and inhalational hydrocarbon-based anaesthetic gases are both recognised to mediate general anaesthesia via the GABA_A_ receptor in the brain, but it is increasingly understood that general anaesthesia is the product of action on many different neuronal receptors rather than via a single mechanism [5]. Both inhalational and TIVA agents have wide-ranging and differing interactions with a host of other molecular targets including potassium and voltage-gated ion channels and glycine receptors [5–7]. One of the suggested benefits of inhalational anaesthesia is suppression of pro-inflammatory mediators that reduce the systemic inflammatory response to the tissue injury caused by surgery [8–10]. In addition, animal studies and observational human studies suggest inhalational anaesthesia may have cardioprotective properties during surgery [11].

There is emerging evidence suggesting anaesthetic techniques may affect disease-free survival amongst patients undergoing cancer surgery. Surgical resection can provide complete removal of primary tumour and potential cure for many cancer patients. However, despite apparently complete tumour resection, disease progression can occur in up to a third of patients. There is some preclinical evidence indicating that the systemic inflammatory response and immune disequilibrium following surgery can allow growth of metastases [12, 13]. Small mechanistic studies have suggested TIVA can prevent immunosuppression and inhibit cancer cell migration [13, 14], but clinical studies have so far not demonstrated any benefit of anaesthetic techniques in cancer outcomes following surgery [15].

Our literature review found 11 systematic reviews and five Cochrane reviews that compared different aspects of intravenous and inhalational anaesthesia. Two systematic reviews which examined the impact of anaesthetic techniques on long-term cancer outcomes suggested with low certainty that TIVA may be beneficial [16, 17]. Two systematic reviews compared complications and mortality of anaesthetic techniques in patients undergoing cardiac surgery and found no differences in peri-operative complications or survival [18, 19]. Eight other systematic reviews compared anaesthetic techniques in selected surgical groups: neurosurgery [20, 21], one lung ventilation in thoracic surgery [22], robotic assisted laparoscopic surgery [23], paediatric surgery [24, 25], ambulatory/day case surgery [25, 26]. The findings suggest TIVA is associated with faster recovery of consciousness, a reduced risk of nausea and vomiting and less pain immediately after surgery [27–29]. A 2018 Cochrane review suggests a reduced risk of post-operative cognitive dysfunction for older patients undergoing non-cardiac surgery with TIVA, but uncertainty remains due to high risk of bias in included studies [30]. Crucially, these systematic reviews did not provide evidence on patient-centred outcomes or safety outcomes such as accidental awareness during anaesthesia. We identified only one large randomised trial in non-cancer patients that compared TIVA with volatile-based anaesthesia in cardiac surgery: the MortalitY in caRdIAc surgery trial (MYRIAD), which included 5400 patients undergoing cardiac surgery who were randomised to TIVA or inhalational anaesthesia. This trial was designed to specifically test whether inhalational anaesthesia was protective against myocardial injury and reduced mortality in patients undergoing cardiac surgery [31]. The trial was stopped early for futility with similar 1-year mortality rates in both groups.

The choice of anaesthesia not only affects the care of patients during surgery but may also impact on their quality of recovery, survival and other patient safety outcomes, including accidental awareness under anaesthesia. Prompt recovery and discharge from hospital will enhance physical recovery, limiting immobility and physical deconditioning [32, 33]. These aspects are of growing importance as frail older patients now undergo major surgery more often than ever before [34, 35].

### Objectives {7}

The primary objective of this trial is to test whether TIVA is superior to inhalational anaesthesia in terms of days alive and at home at 30 days (DAH30), survival and quality of recovery amongst patients undergoing major non-cardiac surgery. Secondary objectives are to evaluate the safety of TIVA, including post-operative complications and incidence of accidental awareness under anaesthesia; and to assess the cost-effectiveness of TIVA.

### Trial design {8}

An open-label multi-centre pragmatic efficient randomised (1:1) superiority trial comparing TIVA and volatile-based anaesthesia in adults aged 50 years and over, undergoing elective major non-cardiac surgery under general anaesthesia with health economic evaluation. The VITAL trial uses an efficient trial design partnering with an existing national cohort study hosted by the Royal College of Anaesthetists: the Perioperative Quality Improvement Programme, PQIP (pqip.org.uk). PQIP has been running successfully since 2017, providing a database of patient outcomes to facilitate benchmarking and quality improvement in perioperative care [36]. VITAL will be nested within PQIP study and utilise existing PQIP database, data collection and follow-up procedures.

Within VITAL, a small qualitative study will be undertaken to examine ways of consenting participants into complementary studies. The study will consist of interviews with participants of VITAL, patients who declined to take part in VITAL and staff members involved in consenting participants into the trial. Potential participants will be contacted by a trained qualitative researcher for their consent to take part in a semi-structured interview exploring their experiences.

## Methods: participants, interventions and outcomes

### Study setting {9}

Patients were under the care of participating surgical and anaesthetic care teams in at least 40 NHS hospitals from England, Wales and Scotland participating in PQIP.

### Eligibility criteria {10}

Participants are eligible to be included in the trial if they meet the following criteria:

Inclusion criteria.

1. Age ≥ 50 years
2. Elective major non-cardiac surgery under general anaesthesia (as per PQIP inclusion criteria)
3. Written informed consent for trial participation

Exclusion criteria.

1. Known contraindication to either TIVA or inhalational anaesthesia
2. Clinician refusal
3. Procedures where the participant is not expected to survive for 30 days
4. Previous participation in VITAL trial
5. Participant unable to give informed consent or complete questionnaires

### Who will take informed consent? {26a}

Potential participants will be screened by research staff at the site having been identified from pre-admission clinic lists, operating theatre lists and by communication with the relevant nursing and medical staff. Before surgery, potential participants will be identified and approached by a member of the research team. This may be conducted via telephone, post, online or face-to-face consultations and provides an opportunity for the research team to explain the trial to the participants in detail. Participant information sheets can be posted or emailed to potential participants for their perusal and consideration. The participant will be approached prior to surgery at the first suitable opportunity to allow time for any questions. It is recommended (although not mandated) that the participant is approached at least 1 day prior to the date of surgery. Written informed consent must be obtained before surgery and can be obtained using either paper or electronic systems depending on individual site arrangements.

### Additional consent provisions for collection and use of participant data and biological specimens {26b}

Not applicable, no biological specimens are collected.

## Interventions

### Explanation for the choice of comparators {6b}

General anaesthesia is most often maintained with an inhaled volatile anaesthetic agent (e.g. sevoflurane, isoflurane) [2]. A commonly used alternative is to maintain anaesthesia using infusions of intravenous anaesthetic drugs (e.g. propofol, remifentanil), a technique termed total intravenous anaesthesia or TIVA. The two techniques have important differences in side effect profile.

## Intervention description {11a}

### Total intravenous anaesthesia (TIVA)

Participants randomised to the TIVA arm of the trial will have their anaesthesia maintained with intravenous anaesthetic agents as determined by the treating anaesthetist. The administration of TIVA will not be protocolised and will be left to clinical discretion for management. Maintenance of general anaesthesia should be via TIVA only. Clinicians are reminded to avoid volatile-based inhalational anaesthetic agents in this participant group.

### Volatile-based inhalational anaesthesia (INH)

Participants randomised to the INH arm of the trial will have their anaesthesia maintained with inhalational volatile-based anaesthetic agents as determined by the treating anaesthetist. Administration of INH will not be protocolised and will be left to clinical discretion for management. Maintenance of general anaesthesia should be via inhalational route only. Clinicians are reminded to avoid intravenous anaesthetic agents in this participant group.

### Criteria for discontinuing or modifying allocated interventions {11b}

It is possible that the participant may receive an anaesthesia technique other than the one that was allocated to them within the trial, for example due to equipment malfunction or change in clinical circumstances. In this pragmatic trial, brief deviations or interruptions in the allocated anaesthesia technique lasting shorter than 20 min will not be interpreted as true cross over between treatment arms.

### Strategies to improve adherence to interventions {11c}

Site teams are trained on trial protocol during site initiation visits and regular updates are provided during site team catch-ups and newsletters. Protocol deviations will be monitored throughout the trial by the trial management group and data safety and monitoring committee. Where deviation rates appear excessive, contact will be made with site investigators to provide retraining and support.

### Relevant concomitant care permitted or prohibited during the trial {11d}

Anaesthesia will be administered by experienced anaesthetists and delivered according to local guidelines. All other participant care will be conducted as per routine clinical practice and information collected in trial case record forms.

### Provisions for post-trial care {30}

The clinical interventions used in the trial are performed at a single point in time and cannot be amended in any way once performed. Both interventions are routinely used in clinical practice. As such, there is no need to provide continuing post-trial care other than as standard local practice. Post-operative complications will be managed as per routine care at participating sites.

## Outcomes {12}

### Effectiveness

#### Primary outcome

The primary outcome is days alive and at home at 30 days after surgery (DAH30). This sensitive measure reflects the number of days from surgery to discharge and also any hospital readmission(s) during that month as well as survival.

#### Secondary outcomes

- ▪ Days alive and at home at 90 days (DAH90)
- ▪ 30-day and 90-day mortalities
- ▪ Six-month mortality
- ▪ Quality of recovery after anaesthesia (QoR-15) at day 3 after surgery [37]
- ▪ Patient satisfaction with anaesthesia (Bauer questionnaire) on day 1 after surgery [38]

### Safety

- Accidental awareness under anaesthesia (modified Brice questionnaire) on day 3 and 30 days after surgery [39]
- Major post-operative complications Clavien-Dindo grade 2 and above within 30 days after surgery [40]

### Health economics

- Health resource use during the 6 months after surgery
- Health-related quality of life evaluated using EuroQoL instrument (EQ-5D-5L) at baseline, at hospital discharge, at 30 days and 6 months after surgery [41, 42]

### Participant timeline {13}

**Table:** 

**Visit window**	**Day 0 Baseline**	**Day 1**^**#**^	**Day 3**^**#**^	**Day of discharge**^**#**^	**Day 30 + 7 days**	**Day 90 + 7 days**	**6 months** ± 21 days
Informed consent	√						
Medical history	√						
Inclusion/exclusion criteria	√						
Surgical speciality	√						
Expected duration of surgery (< 2 h, ≥ 2 h)	√						
Cancer surgery/non-cancer surgery	√						
Preoperative frailty (Rockwood frailty score)	√						
Intervention	√^						
Bauer questionnaire		√^					
QoR-15			√^				
Modified Brice questionnaire			√		√		
Post-operative delirium (4AT)			√				
Post-operative complications (Clavien-Dindo grade II and above)					√^		
Length of stay*				√^			
Survival status*					√^	√	√
Hospital readmission*					√	√	√
Health resource use							√
Quality of life EQ 5D	√^			√^	√^		√^

*Information needed for DAH30 and DAH90

^Already collected by PQIP database

^#^Or closest next working day

### Sample size {14}

The total sample size will be 2500 participants over the two intervention arms (1250 per arm). The primary endpoint of this trial is the number of days alive and at home 30 days after surgery (DAH30). DAH30 is a continuous number between 0 and 30 which reflects, out of the 30 days following surgery, the total number of those days that a participant spends alive and at home [43]. In this definition, home reflects any place other than hospital. If a participant dies within those 30 days, their value is set to 0. The secondary endpoints of this trial include number of days alive and at home 90 days after surgery (DAH90) and survival at 90 days after surgery. The population eligible for entry into VITAL is similar to those participants undergoing elective surgery reported by Bell in 2019 with a mean DAH30 of 25 (SD 6.6) [44]. Applying the VITAL trial inclusion and exclusion criteria to sample data using hospital episode statistics, we found a mean DAH90 of 72.9 (SD 21.3) and a 90-day mortality rate of 3.8%. We have adopted a conservative estimate of 7.5 for the standard deviation of DAH30 and a conservative estimate of 22 for the standard deviation of DAH90. With a 5% two-sided significance level and 90% power, the randomisation of 2500 participants (1:1) to either TIVA or inhalational anaesthesia would allow detection of a difference of 1 day in DAH30 between treatment arms. This sample size allows for up to 5% loss-to-follow-up. Also, with a 5% two-sided significance level, this 2500 participant sample will allow us to detect a difference in DAH90 between arms of 3 days with 90% power, or 4 days, with > 95% power. For a safety non-inferiority analysis of 90-day mortality, assuming there is truly no difference between modes of anaesthesia (3.8% mortality), a 2500 participant sample would provide 80% power for a one-sided 95% confidence interval to exclude a 1.9% increase in mortality due to TIVA (a relative risk of 1.5). A one-sided 97.5% confidence interval would exclude an increase due to TIVA of 2.2% (a relative risk of 1.6).

### Recruitment {15}

Participants will be recruited from at least 40 UK hospital sites with a track record of active and successful recruitment to clinical trials and an appropriate patient case mix.

## Assignment of interventions: allocation

### Sequence generation {16a}

Participants will be randomised on a 1:1 basis to receive either TIVA or inhalational anaesthesia. Randomisation will be undertaken through a simple and secure, Interactive Voice Response Randomisation System (IVRS) that has been established by the programming team at Warwick Clinical Trials Unit. This computerised procedure will use a minimisation algorithm to ensure balance in treatment arm allocation across the following four stratification variables, factors thought to affect outcome either through treatment effectiveness or underlying prognosis, also permitting appropriate exploratory subgroup analyses:

1. Surgical speciality (musculoskeletal/intra-abdominal/thoracic/vascular/other)
2. Expected duration of surgery (< 2 h, ≥ 2 h)
3. Cancer surgery/non-cancer surgery
4. Preoperative frailty (Rockwood frailty score)—well/vulnerable/frail [45]

### Concealment mechanism {16b}

Allocation concealment will be ensured as the IVRS at the WCTU will not release the randomisation code until the patient has been recruited into the trial.

### Implementation {16c}

Recruiting centres will be asked to randomise participants on the day of surgery, once the planned surgery is confirmed as taking place. There will be occasions when surgery is cancelled at the last minute and rescheduled to another day. If the participant is happy to remain in the trial, recruiting centres will be asked to inform the VITAL team at WCTU of the new surgery date as soon as possible, and data collection timepoints will be adjusted. Participants should receive their original allocated intervention at their new scheduled surgery date. If the surgery is cancelled indefinitely, or the participant is no longer suitable for the trial, the recruiting centre will be asked to inform the VITAL team at WCTU as soon as possible.

## Assignment of interventions: blinding

### Who will be blinded {17a}

In this trial, it is not possible to blind participants or the research staff at sites to a participant’s randomised allocation. The primary outcome and most of the clinically reported secondary outcomes are objective. During the trial, the trial management group and the trial steering committee will not see outcome results broken down by treatment arm.

### Procedure for unblinding if needed {17b}

Not applicable; VITAL is an open-label study.

## Data collection and management

### Plans for assessment and collection of outcomes {18a}

Participants will be contacted by telephone at day 30 (+ 7 days) by site research staff to screen for late recall of accidental awareness and collect data on EQ-5D-5L, hospital readmission and any post-operative complications that are classed Clavien-Dindo Severity Grade II or above. Day 90 (+ 7 days) follow-up will be completed by a check of medical records only. Participants will be contacted by telephone at 6 months post-surgery (± 21 days) by site research staff to collect data on health resource use based on participant diary and quality of life using EQ-5D-5L.

Modified Brice Questionnaire is used to screen for recall of accidental awareness under general anaesthesia (AAGA). A potential case of AAGA should be flagged by the local research team if a participant responds that they remembered something between going to sleep and waking up or they answered ‘Awareness’ to the question asking them to report the worst thing about their operation. The principal investigators for each of these participants should be contacted by the local research team and asked to give their opinion of the likelihood of AAGA for their participants as ‘probable’, ‘possible’, ‘unlikely’ or ‘un-assessable’ according to previously defined criteria and using available local data. All cases of probable and possible AAGA should be reported to WCTU by completion of the Brice Questionnaire Additional Data Form. Participants should be followed up locally as per usual care.

### Plans to promote participant retention and complete follow-up {18b}

Throughout the recruitment and follow-up period, retention has been constantly assessed by the trial management group including patient representatives. A participant diary is provided to participants to aid data collection at 6 months and each participant receives a phone call from research team at day 30 and at 6 months. A voucher of nominal value is posted out to participants as a thank you for their participation.

### Data management {19}

Personal data collected during the trial will be handled and stored in accordance with the 2018 Data Protection Act. Participants will be identified using unique trial number only, and no data which identifies participants by name will be shared with nor held at Warwick CTU.

We will use the standard WCTU trial web-based application for data management. Participant data (including case report forms) will be collected in accordance with the protocol from PQIP database. Clinical data will be collected during the hospital stay up to 30 days after randomisation. Baseline characteristics collected include participant demographics, comorbidities, pre-admission function, quality of life, inclusion/exclusion criteria, consent, surgical speciality, expected duration of surgery, time and date of randomisation. Data captured following randomisation will include administered anaesthetic techniques, post-operative complications, participant satisfaction, quality and speed of recovery, accidental awareness, health resource use, health-related quality of life, SAEs and survival status.

The case report form (CRF) has been developed by the WCTU in conjunction with PQIP and made available to the participating sites as a paper and electronic CRF (eCRF) for ease of data collection; supporting materials will be available to staff. On all trial-specific documents, other than the signed consent form, the participant will be referred to by a unique trial-specific number in any database, not by name. Signed consent forms will be retained at the recruiting site and will not be shared with WCTU. The trial will be conducted in accordance with the current approved protocol, Good Clinical Practice (GCP), relevant data protection regulations, the trial data management plan, and WCTU standard operating procedures (SOPs). A monitoring plan and risk assessment will be devised to protect participant safety and integrity of trial data.

### Database

The VITAL database will be developed by the Programming Team at WCTU and will link by unique identifiers to the PQIP database. All specifications (i.e. database variables, validation checks, screens) will be agreed between the programmer and appropriate trial staff. For further information regarding the PQIP database, please refer to PQIP study information (www.pqip.org.uk).

### Data storage

All essential documentation and trial records will be stored by WCTU in conformance with the applicable regulatory requirements, and access to stored information will be restricted to authorised personnel. Any paper data forms will be stored in a lockable filing cabinet in a secure room, to which access is restricted to authorised personnel. Electronic data will be stored in a secure area of the computer with access restricted to staff working on the trial and the WCTU Quality Assurance team. All databases containing identifiable information will be encrypted and password protected. Any data that are transferred out of the secure environment will adhere to WCTU SOPs.

### Data access and quality assurance

All data access will be controlled by individual usernames and passwords, and any changes to data will require the user to enter their username and password as an electronic signature in accordance with regulatory requirements. Staff will have access restricted to the functionality and data that are appropriate for their role in the trial and will not share their log in details.

### Data shared with third parties

Any data transfer would be in accordance with University of Warwick SOPs and require data sharing/processing agreements to be in place prior to transfer.

### Archiving

Trial documentation and data will be archived for at least ten years after completion of the trial. Trial Master File and associated data will be archived by WCTU; trial data generated at sites will be archived according to local policy.

### Confidentiality {27}

The University of Warwick is the Sponsor for the trial. The trial is being conducted in full adherence with the principles of the Declaration of Helsinki and MRC Good Clinical Practice principles and guidelines. It also complies with all applicable UK legislation and Warwick Standard Operating Procedures. All data are being stored securely and held in accordance with the Data Protection Act 2018. All identifiable data are pseudonymised and treated as confidential. All CRFs, questionnaires, trial reports and communication regarding the trial will identify the participants by the assigned unique trial identifier only. Participant confidentiality will be maintained at every stage and identifiable information will not be made publicly available.

### Plans for collection, laboratory evaluation and storage of biological specimens for genetic or molecular analysis in this trial/future use {33}

Not applicable, no biological specimens collected.

## Statistical methods

### Statistical methods for primary and secondary outcomes {20a}

All statistical analyses will be undertaken on an intention to treat basis where possible to preserve randomisation, avoid bias from exclusions and preserve statistical power. Hence, all participants randomised into the trial, regardless of whether they received their randomised intervention, will be analysed according to their randomised group using data collected up to their final follow-up in the trial (6-month timepoint or the last timepoint prior to their withdrawal or loss to follow-up). We will do an additional per-protocol analysis including only participants who received their allocated intervention (as detailed in the statistical analysis plan). Participants not receiving surgery or withdrawing consent for follow-up prior to surgery will not be included in relevant denominators.

For the primary outcome of DAH30, each randomised treatment arm’s point estimate (and 95% confidence interval) will be reported. In addition, DAH30 will be compared across randomised treatment arms using independent samples *t*-tests, Wilcoxon rank sum tests or appropriate modelling techniques depending on the distribution of the data. The secondary outcome of DAH90 will be analysed as per the DAH30 techniques. Rates of mortality and major post-operative complications will be assessed across randomised arms using chi-squared tests, with logistic regression used to adjust for stratification variables.

Quality of recovery, participant satisfaction and accidental awareness will be scored using appropriate manuals.

The four stratification factors used at randomisation define sub-groups of interest.

- Surgical speciality* (intrabdominal/musculoskeletal/thoracic/vascular/other)
- Expected duration of surgery (< 2 h, ≥ 2 h)*
- Cancer surgery/non-cancer surgery*
- Preoperative frailty (Rockwood frailty score*)—well/vulnerable/frail [45]

A Consolidated Standards of Reporting Trials (CONSORT) flow diagram will be produced, showing the frequency of participants:

- Assessed for eligibility
- Excluded prior to randomisation (and the frequency of each reason for exclusion)
- Randomised
- Allocated to each randomisation arm
- Receiving or not receiving their randomised treatment
- Followed-up at each protocol specified timepoint
- Loss to follow-up at each protocol specified timepoint (and the frequency of each reason for loss to follow-up)

### Health economic evaluation

A prospectively planned economic evaluation will be conducted from an NHS and personal social services perspective, according to the recommendations of the NICE reference case [46]. Use of resources during the index admission, where change can be attributed to anaesthesia, will be captured by PQIP. These will include time in surgery and length of stay by level of care. Participants’ community contacts, made in connection with their surgery, will be recorded in the first 6 months. Participants will be encouraged to use an electronic or paper calendar to help recall this information at follow-up. Healthcare resource use will be costed using most recently available published national reference costs, reflated to the most recent year [47]. We will describe reported resource use disaggregated, providing hospital and community usage time horizons. We will simplify resource collection as much as possible, preparing participants to understand the resource information sought and promoting this recording through diaries.

Generic health-related quality-of-life will be assessed at baseline, at discharge, 30 days and at 6 months using the EQ-5D-5L questionnaire. EQ-5D-5L scores will be converted to health status scores using the UK value set recommended by NICE guidance at the time of analysis [48, 49]. Participant-level QALY estimates will be estimated as the area under the curve (AUC) of health status scores over time using the trapezoidal rule. Baseline EQ-5D-5L will be included to minimise bias in the QALY calculation and to adjust subsequent analyses [50, 51]. Whilst a greater number of observations would undoubtedly be desirable, the measurement schedule is necessarily pragmatic within the design of the trial. Varying time to discharge may possibly be a proxy for achieving an adequate quality-of-life. Longer hospital stay increases the contribution of the hospital period to the overall AUC and decreases the contribution of the post-discharge period, as both are time-weighted; hence, the (informatively) varying discharge timepoint should provide a more accurate QALY estimate than a fixed point, because it better characterises the shape of the AUC. We will perform a sensitivity analysis omitting the discharge point as a check for consistency of findings. We will monitor levels of missingness of resource and outcome data, taking steps to promote quality of reporting.

Within-trial analysis (to 6 months) using bivariate regression of costs and QALYs will inform a probabilistic assessment of incremental treatment cost-effectiveness [52]. Mechanisms of missingness of data will be explored and multiple imputation methods will be applied to impute missing data [53–55]. Imputation sets will be used to estimate incremental cost per QALY estimates and confidence intervals. Findings will be analysed and visualised in the cost-effectiveness plane, as cost-effectiveness acceptability curves, net monetary benefit and value of information analysis (EVSI). Should costs and quality-of-life not converge within 6 months, more extensive economic modelling using decision-analytic methods may be considered to extend the target population, time horizon and decision context, drawing on best available information from the literature and stakeholder consultations to supplement the trial data. Parameter uncertainty in the decision-analytic model will be explored using probabilistic sensitivity analysis. If longer term decision modelling is to be undertaken, then costs and outcomes will be discounted at 3.5% after the first year of randomisation in line with NICE reference case [46]. Analyses and modelling will be undertaken in Stata 16 SE (or later release if available). Reporting will follow the Consolidated Health Economic Evaluation Reporting Standards (CHEERS) statement [56].

### Interim analyses {21b}

There are no planned interim analyses.

### Methods for additional analyses (e.g. subgroup analyses) {20b}

Pre-specified sub-group analyses will be undertaken using appropriate modelling techniques. These will be determined following examination of the distributions of the collected data but are anticipated to be linear regression for DAH30 and DAH90 and logistic regression modelling for mortality rates at 90 days. These exploratory sub-group analyses will have lower power than the main whole trial analysis but are hypothesis-generating, and results will be scrutinised graphically via forest plots.

### Methods in analysis to handle protocol non-adherence and any statistical methods to handle missing data {20c}

Every effort will be made to collect full baseline, treatment and follow-up data on all trial participants; it is thus anticipated that missing data will be minimal. Participants with missing primary outcome data will not be included in the primary analysis in the first instance. This presents a risk of bias, and sensitivity analyses will be undertaken to assess the possible impact of the risk. This will consist of simulating the missing responses using a multiple imputation approach.

### Plans to give access to the full protocol, participant-level data and statistical code {31c}

The full protocol is publicly assessable online via the trial website. Data collected within the VITAL study will be made available to researchers whose full proposal for their use of the data has been approved by the VITAL trial management group and whose research group includes a qualified statistician. The data required for the approved, specified purposes and the trial protocol will be provided, after completion of a data sharing agreement. Data sharing agreements will be set up by the sponsors of the trial, the funders, the trial coordination centre and the trial steering and management groups.

## Oversight and monitoring

### Composition of the coordinating centre and trial steering committee {5d}

The trial is managed by a multi-disciplinary team. Trial management will be based at WCTU, University of Warwick. All day-to-day management of the trial will be the responsibility of the CIs, Dr Joyce Yeung and Dr Shaman Jhanji, with tasks delegated to appropriate members of the trial management team.

The trial management team will assist and facilitate the setting up of centres wishing to collaborate in the trial. In addition, the trial management team will:

- Set up standardised database access for collaborators
- Organise the telephone randomisation service for formal trial entry
- Monitor the collection of data, process data and seek missing data
- Train local staff with regards to data collection remotely
- Ensure the confidentiality and security of all trial forms and data
- Conduct extensive data checking and cleaning
- Organise the trial analyses
- Organise and create reports for steering committee, DMC and collaborator meetings

The trial management team will receive data downloaded from the PQIP database. Upon receipt, data forms will be checked for completeness and entered into a trial-specific dedicated computer programme.

### Trial management group (TMG)

The trial management group, consisting of the project staff and co-investigators involved in the day-to-day running of the trial, will meet regularly throughout the project. Significant issues arising from management meetings will be referred to the trial steering committee, investigators or funder, as appropriate.

### Trial steering committee (TSC)

The trial will be guided by a group of respected and experienced trialists as well as one ‘lay’ representatives. The TSC will have an independent chairperson. Face to face or online meetings will be held at regular intervals determined by need but not less than once a year. Routine business is conducted by email, post or teleconferencing.

The steering committee, in the development of this protocol and throughout the trial, will take responsibility for:

- Major decisions such as a need to change the protocol for any reason
- Monitoring and supervising the progress of the trial
- Reviewing relevant information from other sources
- Considering recommendations from the DMC
- Informing and advising on all aspects of the trial

The full remit and responsibilities of the TSC will be documented in the committee charter which will be signed by all members.

### Composition of the data monitoring committee, its role and reporting structure {21a}

#### Data monitoring committee (DMC)

A DMC will be appointed comprising of two independent clinicians with experience in clinical trials and an independent statistician. One of the independent clinicians will have experience in undertaking clinical trials in emergency or acute care.

The roles of the DMC will include:

- Monitoring the data and making recommendations to the TSC on whether there are any ethical or safety reasons why the trial should not continue
- Advising the TSC regarding the release of data and/or information
- Considering data emerging from other related studies

It is anticipated that the DMC members will meet once prior to the commencement of the trial to agree the committee charter, once at the end of the 6-month pilot, with subsequent meetings throughout the course of the trial. DMC meetings will also be attended by the chief investigator and trial manager/coordinator (for non-confidential parts of the meeting) and the trial statistician(s). The full remit and responsibilities of the DMC will be documented in the committee charter which will be signed by all members.

### Adverse event reporting and harms {22}

VITAL is a non-CTIMP trial and all trial interventions are already in routine clinical use for participants undergoing major non-cardiac surgery. Expected post-operative complications (starting from end of surgery on day of surgery to 30 days post-surgery) will be collected as a trial outcome and will not be recorded separately as AEs. These events will be included as part of the safety analysis for the trial and therefore do not need to be reported separately to the trial coordinating centre. No additional AEs will be collected, other than those specified as post-operative complications (Additional file 1: Appendix). Post-operative complications will be reviewed/monitored at intervals by the DMC.

Beyond these defined, expected complications, adverse events will be reported in accordance with the UK Policy Framework for Health and Social Care Research, the Principles of GCP as set out in the UK Statutory Instrument (2004/1031; and subsequent amendments) and the requirements of the Health Research Authority (HRA). When completing an SAE form, the principal investigator or medically qualified delegate will be asked to define the causality and the severity of the SAE. On receipt of an SAE form at the trial office, the chief investigator or delegate will independently determine causality of the SAE. An SAE judged by the PI, CI or delegate(s) to have a reasonable causal relationship with the intervention will be regarded as a related SAE. The CI or delegate(s) will assess all related SAEs for expectedness. If the event is unexpected, i.e. is not defined in the protocol as an expected event, it will be classified as an unexpected and related SAE.

### Frequency and plans for auditing trial conduct {23}

VITAL is a non-CTIMP which has been formally risk assessed by the sponsor as ‘low risk’ on the basis that both interventions are already in common usage throughout the UK, and the safety profiles are well established. A risk-based proportionate approach outlined in the monitoring plan has been developed through discussion with the trial sponsor. It is anticipated that monitoring activity will be predominantly central and remote.

### Plans for communicating important protocol amendments to relevant parties (e.g. trial participants, ethical committees) {25}

All study (including protocol) amendments will be submitted for approval to the REC and HRA. Sites will be informed of all approved minor or substantial amendments and will be asked to review and confirm approval at local site level.

### Dissemination plans {31a}

Data arising from this research will be made available to the scientific community in a timely and responsible manner. The main scientific report will be drafted by senior investigators on behalf of VITAL trial group in accordance with the Consolidated Standards of Reporting Trials (CONSORT) guidelines (www.consort-statement.org). VITAL Publication and Dissemination Working Party will agree the membership of a Writing Group Committee, which will take primary responsibility for final data analysis and writing of the scientific report.

The results of the trial will be shared widely, and participants are able to request a copy of the results through contacting the local trial team. Patient research partners will help the production of a plain English summary of trial results which will be produced to aid patients and the public in understanding the options and differences in anaesthetic techniques and to consider their preferences. Following the conclusion of the trial, summary information will be made available to patients and the public via trial website. A video and/ or infographic to communicate trial results to the public will be produced with the support of our PPI research partners.

## Discussion

The 7th National Audit Project published in 2023 reported a dramatic increase in proportion of general anaesthetic cases using TIVA or propofol as maintenance agent from 8% in 2013 to 26% in 2023, representing an increase of more than threefold [57]. The reasons behind this significant change in practice may be a combination of environmental concerns, perceived benefits in cancer recurrence and the accessibility of equipment. The lack of data to support the choice of general anaesthesia has never been more evident.

Patients presenting for surgery are now older, more likely to be overweight and with more co-morbidities [57]. As result, surgical patients are becoming more challenging for anaesthetists and more patients are at risk of complications. Given the very large number of patients exposed to general anaesthesia every year, even small differences in outcome between the two techniques could result in substantial excess harm. Our randomised trial will quantify the benefits and harms of each technique in terms of patient recovery, survival and safety.

The lingering impact of the COVID-19 pandemic disruption on healthcare provision continues to impact on the number of patients on waiting list [58]. Getting patients safely and quickly through their operative journey will also help the recovery of our healthcare services. Results from VITAL study will ensure patients can benefit from the very safest anaesthesia care, promoting an early return home, reducing healthcare costs and maximising the health benefits of surgical treatments.

## Trial status

VITAL protocol v3.0 10 August 2023. Recruitment began on January 2022; recruitment was completed on 10 April 2024.

### Supplementary Information

Additional file 1: Appendix. VITAL Post operative complications outcome definitions.

## Data Availability

The final data set itself will only be available to the direct VITAL trial team, including the TSC, in the first instance. It will however also be made available upon formal request when the reason for the request is approved by the TSC.

## Abbreviations

AAGA: Accidental awareness under general anaesthesia
AUC: Area-under-the-curve
DAH30: Days alive and at home at 30 days
DAH90: Days alive and at home at 90 days
DMC: Data monitoring committee
GABA: γ-Aminobutyric acid type A
GCP: Good Clinical Practice
HRA: Health Research Authority
INH: Inhalational/volatile-based anaesthesia
NICE: National Institute of Health and Care Excellence
non-CTIMP: Trials that do not involve an Investigational Medicinal Product
PPCs: Postoperative pulmonary complications
PPI: Patient and public involvement
PQIP: The Perioperative Quality Improvement Programme
QALY: Quality-adjusted life year
QoR: Quality of Recovery
REC: Research ethics committee
SOP: Standard operating procedure
TIVA: Total intravenous anaesthesia
TMG: Trial management group
TSC: Trial steering committee
WCTU: Warwick Clinical Trials Unit
