# Supplementary material for: Volatile vs Total intravenous Anaesthesia for major non-cardiac surgery: a pragmatic randomised triaL (VITAL)
Source: Trials. 2024 Jun 27;25:414. doi: 10.1186/s13063-024-08159-w (PMC11210167; doi:10.1186/s13063-024-08159-w)
Supplement: Supplementary file 1 — Additional file 1: Appendix. VITAL Post operative complications outcome definitions. [file 13063_2024_8159_MOESM1_ESM.docx]

## APPENDIX: VITAL Post operative complications outcome definitions

**1. Acute Cardiac events**

*Myocardial infarction**

Acute myocardial injury with clinical evidence of acute myocardial ischaemia and with detection of an increase or decrease in cardiac Troponin values with at least one value above the 99th percentile Upper Reference Limit and at least one of the following:

- - 1. Symptoms of myocardial ischaemia
    2. New ischaemic ECG changes
    3. Development of pathological Q waves
    4. Imaging evidence of new loss of viable myocardium or new regional wall motion abnormality in a pattern consistent with an ischaemic aetiology
    5. Identification of a coronary thrombus by angiography or autopsy

*Non-fatal cardiac arrest**

Successful resuscitation from either documented or presumed ventricular fibrillation, sustained ventricular tachycardia, asystole, or pulseless electrical activity

requiring cardiopulmonary resuscitation, pharmacological therapy, or cardiac defibrillation

*Cardiac death**

Death with a vascular cause and includes deaths after a myocardial infarction, cardiac arrest and cardiac revascularization procedure

*Coronary revascularization**

Cardiac revascularisation procedure including percutaneous coronary intervention or coronary artery bypass graft surgery within 30 days of the index surgery

** Any of these outcomes will also count as Major adverse cardiac event (MACE)*

*Atrial fibrillation*

New onset of irregularly irregular heart rate in the absence of P waves lasting at least 30 s or for the duration of the ECG recording (if <30 seconds)

*Use of vasopressor/inotropic support by infusion post-surgery*

*Deep venous thrombosis*

Diagnosis of deep venous thrombosis required any one of the following:

- - 1. A persistent intraluminal filling defect on contrast venography
    2. Non-compressibility of one or more venous segments on B mode compression ultrasonography
    3. A clearly defined intraluminal filling defect on contrast enhanced CT

*Pulmonary embolism*

Diagnosis of pulmonary embolism requires any one of the following:

1. A high probability ventilation/ perfusion lung scan
2. An intraluminal filling defect of segmental or larger artery on a helical CT scan
3. An intraluminal filling defect on pulmonary angiography
4. A positive diagnostic test for deep venous thrombosis (e.g. positive compression ultrasound) and one of the following:
   1. Non-diagnostic (i.e. low or intermediate probability) ventilation/perfusion lung scan
   2. Non-diagnostic (i.e. subsegmental defects or technically inadequate study) helical CT scan

**2. Acute Kidney Injury Stage 3**

According to the KIDGO consensus definition of acute kidney injury (2012): Serum Creatinine 3.0 times baseline OR ≥4.0 mg/dl (≥353.6 mmol/l) increase OR Initiation of renal replacement therapy AND/OR Urine Output <0.3ml/kg/hr for ≥ 24 hours OR No urine output ≥ 12 hours

**3. Infective complications**

*Fever*

Core body temperature >38.5 more than 24 hours following surgery with two readings within a 12-hour period

*Clinical suspicion of infection and antibiotic use other than prophylaxis*

Suspected site: Chest/Urinary/Blood/Wound/Other

**4. Post-operative Pulmonary complications**

*** Any of the outcomes will also count as Post-operative pulmonary complications*

Exclusions: pulmonary embolism, pleural effusion, cardiogenic pulmonary oedema, pneumothorax and bronchospasm.

*Atelectasis***

Diagnosis on computed tomography or chest radiograph

*Pneumonia***

Two or more serial chest radiographs with at least one of the following features (one

radiograph is sufficient for patients with no underlying pulmonary or cardiac disease):

new or progressive and persistent infiltrate

consolidation

cavitation

AND at least one of the following:

1. fever (>38°C) with no other recognised cause
2. leucopaenia (< 4 x 109/L) or leucocytosis (>12 x 109/L)
3. for adults >70 years old altered mental status with no other cause

AND at least two of the following:

1. new onset of purulent sputum or change in character of sputum or increased respiratory secretions or increased suctioning requirements
2. new onset or worsening cough or dyspnoea, or tachypnoea
3. rales or bronchial breath sounds
4. worsening gas exchange (hypoxia, increased oxygen requirement, increased ventilator demand)

*Acute respiratory distress syndrome***

According to the Berlin consensus criteria (2012):

1. Within one week of a known clinical insult or new worsening respiratory symptoms
2. AND bilateral opacities on chest imaging, not fully explained by effusions, lobar/lung collapse, or nodules
3. AND respiratory failure not explained by cardiac failure or fluid overload (requires objective assessment e.g. echocardiogram to exclude hydrostatic oedema if no risk factors are present)
4. AND supplemental oxygenation (requires correcting if altitude >1000m):

- Mild: PaO2:FiO2 26.7-40.0 kPa with PEEP or CPAP ≥ 5cmH2O
- Moderate: PaO2:FiO2 13.3-26.6 kPa with PEEP ≥ 5cmH2O
- Severe: PaO2:FiO2 ≤ 13.3 kPa with PEEP ≥ 5cmH2O

*Pulmonary aspiration***

Diagnosis by clear clinical history AND radiological evidence

Severity:

- Mild: therapeutic supplemental oxygen <0.6 FiO2
- Moderate: therapeutic supplemental oxygen <0.6 FiO2, requirement for high-flow nasal oxygen, or both
- Severe: unplanned non-invasive mechanical ventilation, CPAP, or invasive mechanical ventilation requiring tracheal intubation

**6. Stroke**

Cerebral infarction or intracerebral haemorrhage on computed tomography or magnetic resonance imaging scan, or new neurological signs (paralysis, weakness, or speech difficulties) lasting >24 hours or leading to earlier death.
